# Garnet, the archetypal cubic mineral, grows tetragonal

**DOI:** 10.1038/s41598-019-51214-9

**Published:** 2019-10-11

**Authors:** B. Cesare, F. Nestola, T. Johnson, E. Mugnaioli, G. Della Ventura, L. Peruzzo, O. Bartoli, C. Viti, T. Erickson

**Affiliations:** 1 Dipartimento di Geoscienze, Università degli Studi di Padova, via Gradenigo 6, 35131 Padova, Italy; 2 School of Earth and Planetary Sciences, Curtin University, Bentley, 6102 Perth Australia; 3 Center for Nanotechnology Innovation@NEST, Istituto Italiano di Tecnologia, Piazza San Silvestro 12, 56127 Pisa, Italy; 4 Dipartimento di Scienze, Università di Roma Tre, Largo San Leonardo Murialdo 1, 00146 Rome, Italy; 5 Istituto Nazionale di Fisica Nucleare, Via Enrico Fermi 40, 00044 Frascati, Italy; 6 Istituto di Geoscienze e Georisorse, CNR, via Gradenigo 6, 35131 Padova, Italy; 7 Dipartimento di Scienze Fisiche, della Terra e dell’Ambiente, Università di Siena, 53100, Siena, Italy; 8 Jacobs – JETS, NASA Johnson Space Center, Astromaterials Research and Exploration Science Division, Mailcode XI3, 2101 NASA Parkway, Houston, TX 77058 USA

**Keywords:** Mineralogy, Petrology

## Abstract

Garnet is the archetypal cubic mineral, occurring in a wide variety of rock types in Earth’s crust and upper mantle. Owing to its prevalence, durability and compositional diversity, garnet is used to investigate a broad range of geological processes. Although birefringence is a characteristic feature of rare Ca–Fe^3+^ garnet and Ca-rich hydrous garnet, the optical anisotropy that has occasionally been documented in common (that is, anhydrous Ca–Fe^2+^–Mg–Mn) garnet is generally attributed to internal strain of the cubic structure. Here we show that common garnet with a non-cubic (tetragonal) crystal structure is much more widespread than previously thought, occurring in low-temperature, high-pressure metamorphosed basalts (blueschists) from subduction zones and in low-grade metamorphosed mudstones (phyllites and schists) from orogenic belts. Indeed, a non-cubic symmetry appears to be typical of common garnet that forms at low temperatures (<450 °C), where it has a characteristic Fe–Ca-rich composition with very low Mg contents. We propose that, in most cases, garnet does not initially grow cubic. Our discovery indicates that the crystal chemistry and thermodynamic properties of garnet at low-temperature need to be re-assessed, with potential consequences for the application of garnet as an investigative tool in a broad range of geological environments.

## Introduction

Garnet is one of the most widely occurring minerals in the Earth. It is stable to temperatures (*T*) approaching 2000 °C and pressures (*P*) of ~25 GPa, and occurs in a broad variety of rock compositions ranging from mantle peridotite to metamorphosed basalt, granite and mudstone^1^. Owing to its prevalence, durability and compositional diversity, including the ability to preferentially incorporate particular trace elements and isotopes, garnet is one of the most useful minerals for investigating a wide range of fundamental geological processes. These include estimating the *P–T* evolution and oxygen fugacity of rocks^2–5^, constraining volatile fluxes in the crust and mantle^6,7^, determining the absolute timing and rates of geological processes^8,9^, assessing the rheological properties of the lithosphere^10^, constraining the geodynamic setting of magmatic and metamorphic systems^11,12^, and tracking individual earthquake cycles^13^.

Garnet has the general formula X_3_Y_2_(SiO_4_)_3_^14,15^. In almost all metamorphosed crustal rocks in which it occurs, the composition of garnet lies between the end members pyrope [Mg_3_Al_2_(SiO_4_)_3_], almandine [Fe^2+^_3_Al_2_(SiO_4_)_3_], spessartine [Mn_3_Al_2_(SiO_4_)_3_] and grossular [Ca_3_Al_2_(SiO_4_)_3_)]^16^. Such ‘common’ garnet, which is anhydrous, typically has a cubic structure (space group *Ia*-3*d*) and is optically isotropic^15^. Much rarer is so-called grandite garnet, a solid solution between grossular and andradite [Ca_3_Fe^3+^_2_(SiO_4_)_3_], and hydrogrossular garnet [Ca_3_Al_2_(SiO_4_)_3−x_(H_4_O_4_)_x_]. These unusual compositions typically exhibit optical birefringence that is accompanied by oscillatory or sector zoning. In these cases, the birefringence is either related to a departure from cubic symmetry^17,18^, or to intergrowths with structural mismatches that induce lattice strain^19^.

Birefringence has rarely been noted in common garnet^20^. In such cases, the anisotropy has been attributed to either externally-imposed strain or internal lattice strain, the latter due to the size difference between larger Ca cations and smaller Fe, Mg or Mn cations in the *X* site of the structure^20^. In one case, this mismatch has been interpreted to produce partial long-range ordering and, based on X-ray single-crystal diffraction, a tetragonal symmetry has been proposed for a common garnet from an eclogite^21^. For this sample, however, the differences in the refined unit-cell parameters were too small to unambiguously demonstrate a non-cubic symmetry.

Here we demonstrate that the commonly accepted view that a tetragonal symmetry is restricted to some grandite and hydro- (or fluoro-) garnet compositions^15^ is wrong. Using some spectacular natural samples of garnet-bearing high-*P*, low-*T* metamorphosed basalt (blueschist) exhumed from subduction zones and greenschist-facies metamorphosed mudstones (phyllites) from the roots of mountain ranges, and utilising a multi-technique approach including optical microstructural analysis, BSEM, EMPA, EBSD, FTIR, TEM and single-crystal XRD, we show that common garnet in these low-*T* regional metamorphic rocks^16^ initially grows as a tetragonal, not cubic, mineral.

## Results

### Petrography of tetragonal garnet

**Figure 1:**
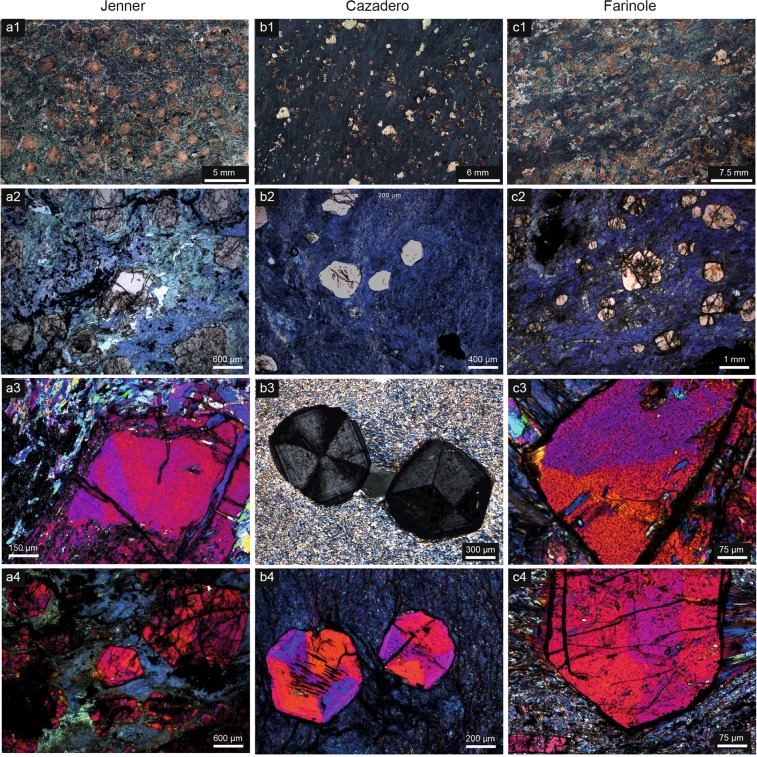
Macroscopic views and petrographic features of the studied blueschists, with emphasis on optical anisotropy of garnets. (**a**) Jenner; (**b**) Cazadero; (**c**) Farinole.

**Figure 2:**
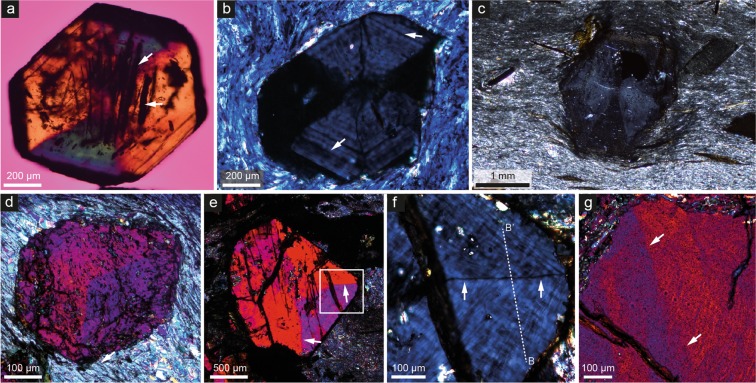
Typical examples of birefringence in the studied garnets. (**a**) 200-μm-thick wafer of an isolated euhedral crystal from Cazadero, California, with sector zoning defined by three pairs of opposite sectors. Arrows point to inclusions of riebeckite. Crossed polarizers (XP) and lambda plate (λ). (**b**) Regular 30-μm thin section view of a partly resorbed garnet set in a fine-grained matrix of Na-amphibole. Arrows point to the subtle concentric oscillatory zoning. Cazadero, XP. (**c**) Optically sector-zoned garnet porphyroblast in a biotite-graphite schist from Pfitscher Joch. 100-μm-thick section, XP. (**d**) Optically sector-zoned garnet in a chlorite-muscovite phyllite from the eastern Alps. 100-μm-thick section, XP, λ. (**e**) 30-μm thin section view of a sector-zoned garnet in a blueschist from Farinole, Corsica. Arrows indicate the boundaries between sectors. White square indicates area enlarged in (**f**). XP, λ. (**f**) Detail of (**e**) showing the mottled pattern of birefringence within sectors. Arrow indicates the sharp line, that is not a microfracture, corresponding to the sector boundary. XP. The B-B’ line corresponds to the EMP transect reported in Fig. 3c. (**g**) Detail of a garnet in a blueschist from Jenner, California showing a well-developed mottled pattern. Arrows indicate a poorly defined sector boundary. XP, λ.

Garnet exhibiting optical birefringence is common in samples of blueschist from the Franciscan mélange, California^22^ (specifically at the Cazadero and Jenner localities) and from Corsica (the Marine de Farinole locality) and in phyllites and micaschists from the central and eastern Italian Alps (namely at Maniva pass, Pfitscher Joch and a third unspecified locality) (Figs 1 and 2; Supplementary Fig. S1, Supplementary Videos S1–S4). The Cazadero blueschist has a very simple mineralogy, consisting of sodic amphibole, garnet, pyrite and quartz, with accessory apatite and allanite. Garnet porphyroblasts form euhedral to partly resorbed crystals up to 1.5 mm in diameter, which have inclusion-rich cores. The blueschist from Jenner contains euhedral, fractured porphyroblasts of garnet up to 3 mm in diameter, set in a weakly-foliated matrix composed mainly of glaucophane with minor quartz, chlorite, phengite and titanite. At Farinole, Corsica, birefringent garnet is associated with glaucophane, zoisite, relict omphacite, rutile, ilmenite, titanite and retrograde chlorite. The garnet is euhedral, up to 3 mm in diameter, commonly fractured, and may contain inclusions of glaucophane. The sample from Maniva pass is a fine-grained muscovite–chlorite–albite–quartz phyllite, with scarce biotite and abundant garnet <0.4 mm in diameter that is partially replaced by chlorite. The sample from Pfitscher Joch is a biotite–muscovite–chlorite schist with euhedral garnet porphyroblasts up to 3 mm in diameter. It contains epidote but no plagioclase. The sample from the eastern Alps is a chlorite-rich, biotite-free, muscovite–albite phyllite containing euhedral garnet (<3 mm across) that shows only very limited replacement by chlorite.

The Cazadero blueschists equilibrated at temperatures of less than 350 °C and pressures between 0.5 and 0.9 GPa^23,24^. The studied sample lacks omphacite, and it is likely that these rocks never experienced higher temperatures^25^. Given the anticlockwise metamorphic evolution proposed for the Franciscan rocks in the area^26^, the Jenner blueschists probably record temperatures close to 400 °C and pressures in excess of 1.0 GPa. The Farinole blueschists record temperatures of 400–500 °C and pressures of 0.7–0.9 GPa during a blueschist-facies event that postdated eclogite-facies metamorphism^27^. The mineral assemblage of the phyllites from Maniva and the eastern Alps suggests metamorphic conditions in the lower greenschist facies (*T* < 450 °C). Conversely, the schist from Pfitscher Joch reached lower amphibolite-facies conditions of 520 ± 30 °C and 0.65 ± 0.1 GPa^28^.

In the Cazadero sample, birefringence is apparent in conventional 30 μm thin sections in crossed polarised light, but in the samples from the other cases it is so weak that it is easily overlooked. In those instances, the use of thicker sections (100 μm) reveals patterns of birefringence (Fig. 2a. Supplementary Fig. S1). Sector zoning^29^ is the most striking optical evidence of birefringence (Figs 1 and 2a–d; Supplementary Fig. S2 and Videos S1–S3), which is most clearly developed in garnet from the Cazadero blueschist^30^ and in the phyllites from the eastern Alps and Pfitscher Joch. The sector zoning appears to follow a rhombdodecahedral or combined icositetrahedral–rhombdodecahedral pattern^31^. The boundary between the sectors is sharp and outlined by a thin black line in crossed polarized light (Figs 1 and 2e, f), but is invisible in plane polarized light.

Irregular mottled birefringence is additionally developed in garnets that show sector zoning, but also in grains that do not. The best examples, preserved in the Jenner and Farinole blueschists and in the Pfitscher Joch micaschist, comprise thin stripes (layers in three dimensions) with a thickness up to a few tens of micrometers. The stripes are either straight or slightly curved and anastomosing, and are typically arranged in two orientations that intersect at a high angle (Fig. 2f,g, Supplementary Fig. S1, Supplementary Video S4). A third mode of optical birefringence is evident as thin concentric oscillatory zoning, which occurs in garnets within the Cazadero blueschist (Figs 1 and 2b).

### The chemical composition of tetragonal garnet

**Figure 3:**
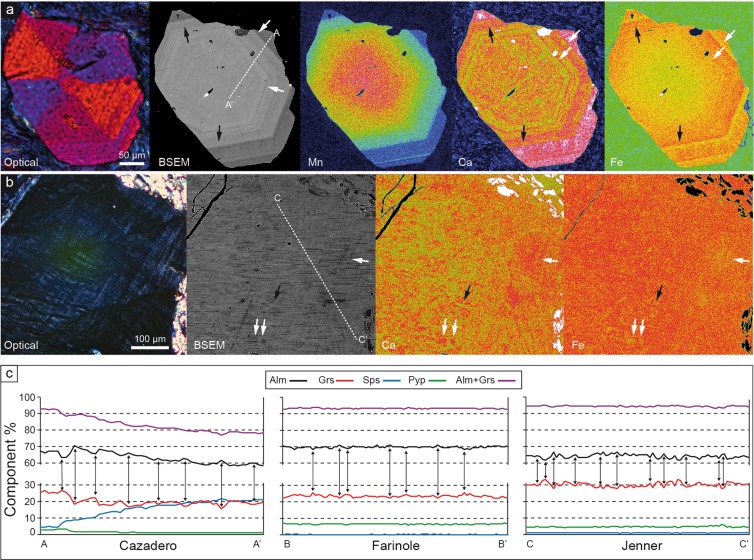
Compositional patterns and inhomogeneities in the studied garnets. (**a**) Series of images showing, from left to right, optical and BSEM view of a sector-zoned garnet from Cazadero, followed by the X-ray maps with the distribution of Mn, Ca and Fe. White arrows point to Ca-high, Fe-low layers, black arrows to Ca-low, Fe high layers in the oscillatory-zoned part of the crystal. The A-A’ line corresponds to the EMP transect reported in (**c**). (**b**) Details of a zone with marked mottled birefringence in a garnet from Jenner. From left to right optical and BSEM views, Ca and Fe X-ray maps of the same area. Arrows like in (**a**). The C-C’ line corresponds to the EMP transect reported in (**c**). (**c**) Major element compositional features in crystals of blueschists along transects in Figs 1f and 3a,b. A-A’: Cazadero: transect from rim (A) to core (A’) of crystal. The features related to oscillatory zoning are apparent on the left part of profile. B-B’: Farinole and C-C’: Jenner. Details of the bands and patches of Ca-Fe variations. Peaks and troughs have maximum widths of 10–20 μm. For all transects the vertical arrows locate the counterbalancing variations of Ca and Fe mirroring each other.

The major element chemical composition of the garnets varies among samples, with most showing core–rim zonation (Fig. 3; Supplementary Fig. S2 and Table S1). In all samples, garnet is dominated by the almandine component (>58 mol.%) and contains significant amount of grossular (18–33%), variable spessartine (<22%) and very low pyrope (1–9%). Only the rim of the Pfitscher Joch sample departs from this compositional range, containing only 12 mol% grossular and 11 mol.% pyrope. A pronounced to weak bell-shaped distribution of Mn, in particular in the Cazadero and Farinole blueschists (Fig. 3a; Supplementary Fig. S2), is consistent with the preservation of growth zoning, and these garnets also preserve concentric oscillatory zoning towards the rim. Comparison of the patterns of chemical zoning and optical sector zoning reveals that the two are unrelated–that is, the boundaries of the optical sectors do not correspond to chemical discontinuities.

**Figure 4:**
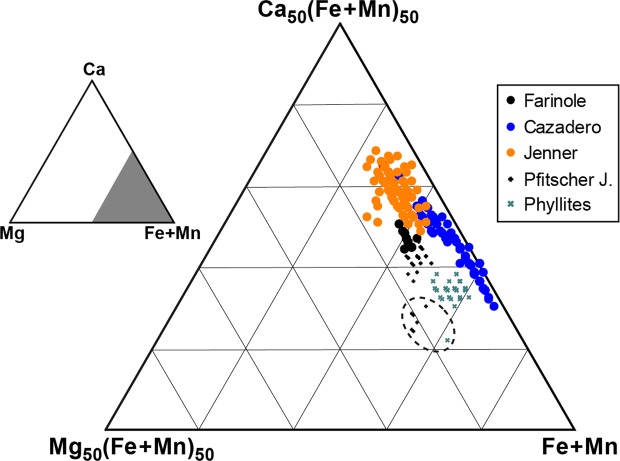
Triangular compositional plot of all EMP garnet analyses. Dashed ellipse marks the compositions of the outermost rims of Pfitscher Joch micaschist and of Maniva Pass phyllite, characterized by lower Ca and higher Mg contents.

The oscillatory zoning at the rims of garnet involves primarily Fe^2+^ and Ca (Fig. 3a,c) that vary antithetically by up to 5 mol.% whereby the combined concentration of almandine and grossular remains constant. Most importantly, direct exchange of Ca for Fe^2+^ is also observed in portions of crystals devoid of optical oscillatory zoning, appearing as zones of mottled birefringence (Fig. 3b,c). Chemical profiles across these zones (Fig. 3c) indicate an almost perfect antithetic coupling between Ca and Fe^2+^, in which concentrations of these components vary by up to 3 mol.% over distances of up to a few tens of micrometres. This suggests that the mottled birefringence may be related to an inhomogeneous distribution of Ca and Fe. The same pattern of antithetic variations in Ca and Fe^2+^ is observed also in the core of a garnet from the Cazadero blueschist. The oscillatory zoning observed at Cazadero, involving essentially a CaFe_−1_ exchange, is different from the rhythmic zoning in Mg and Mn that is commonly developed in other garnets from subduction zone environments and which is considered to track changes in pressure, in some cases reflecting earthquakes cycles^13^. In the blueschist garnets studied here, the Mg content is constant and very low. The birefringent garnet grains have compositions peculiar to blueschist-facies rocks^16^ and very low-grade phyllites^32,33^, defining a narrow compositional field with the lowest Mg/Ca values measured in metasedimentary and metabasic rocks (Fig. 4).

**Figure 5:**
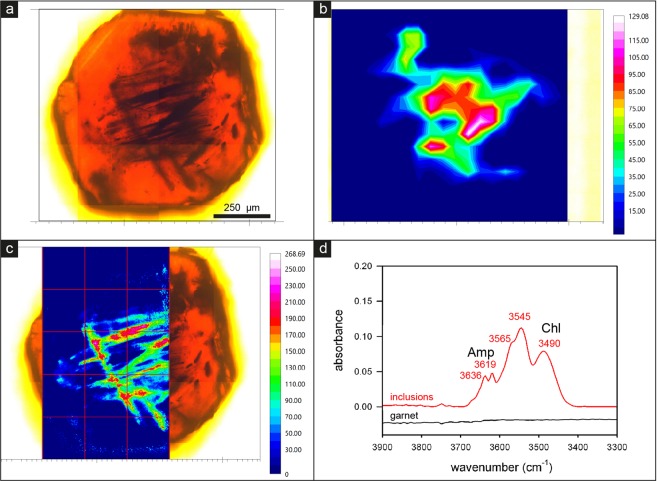
FTIR imaging and the distribution of the hydrous components in a garnet from Cazadero. (**a**) Optical image of the examined sample. (**b**) Distribution of the hydrous components resulting from a grid of 40 × 40 μm^2^ single spots. (**c**) High-resolution image collected as a grid (reported on the image) of 15 FPA spots, each covering 170 × 170 μm^2^; the image clearly shows that the hydrous components are strictly related to the included fibrous minerals, while the garnet host is anhydrous. Both images were obtained by integrating the signal in the OH-stretching 3700–3400 cm^−1^ range. The intensity of the absorption is proportional to the colour scale on the left, where blue = zero and red = maximum. (**d**) Selected single spectra (plotted with the same absorbance scale), collected with a 40 × 40 μm beam in a fibrous-rich area toward the crystal core (red line), and in a clean area in the garnet host (black line). The spectrum collected in the garnet host is totally flat, indicating the sample to be totally anhydrous; the spectrum collected in the hydrous zone shows a convoluted absorption (see Methods); the peaks of amphibole (Amp) and phyllosilicate (Chl, a phase close in composition to a chlorite) are evidenced. Spectra collected in the NIR (6000–4000 cm^−1^) range in the hydrated core (not shown) display only a weak band at 4170 cm^−1^ while no absorption occurs at wavenumbers >5000 cm^−1^; this indicates the presence of OH-groups only as hydrous component in the sample.

As birefringence in garnet may be related to the presence of OH or H_2_O in the structure^14^, we analysed the sector-zoned garnets from Cazadero, the eastern Alps and Pfitscher Joch by FTIR imaging, collecting both single-spots maps and FPA (focal-plane-array of detectors) images (Fig. 5). The results show that, although garnet in the Cazadero sample contains 100–300 μm-long needles and lamellae of hydroxylated minerals including chlorite, deerite, stilpnomelane, and phengite, the garnet itself is anhydrous within the limits of the technique (few ppm; Fig. 5c). Similarly, in the two phyllite samples, the garnet is OH-free, and the OH signal in the FTIR images is clearly associated with inclusions of phyllosilicates (Supplementary Fig. S3).

### Constraints on crystal structure

Garnet crystals from five samples were characterized by single-crystal X-ray diffraction to collect complete intensity and structural data (see Methods). In all samples the systematic absences and the statistical analysis of the intensities are consistent with a tetragonal structure with *I*4_1_/*acd* space group (Supplementary Table S2). The difference between a,b and c cell edges is 17 to 35 times the experimental uncertainty. In the sample from Pfitscher Joch this difference is smaller, but still five times larger than the uncertainty.

A birefringent garnet from the Farinole blueschist was also investigated by an alternative single-crystal XRD technique that is both more precise and more accurate (see Methods). The data (45 different reflections each measured in eight different positions) confirm the tetragonal symmetry with *a* = *b* = 11.6064(4) Å, *c* = 11.6146(4) Å, and a unit cell volume of 1564.59(14) Å^3^, in which the difference between *a*, *b* and *c* edges is more than ten times the experimental uncertainty (Supplementary Table S2). The XRD data do not indicate any site preference among Fe, Ca, Mn and Mg within the X1 and X2 sites.

**Figure 6:**
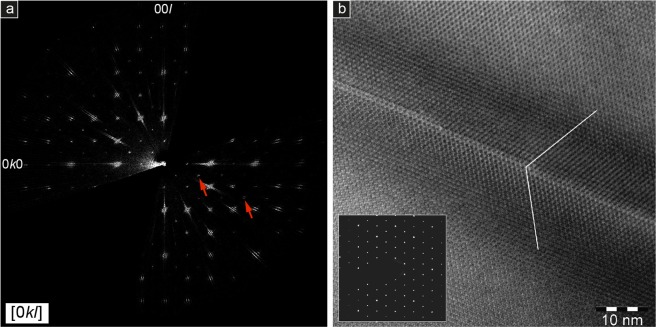
Structural TEM features of garnets from Cazadero. (**a**) Section of the 3D diffraction volume obtained by EDT data: 0kl plane, showing reflections 0*kl*: *k*, *l* ≠ 2*n*, not consistent with a *Ia*-3*d* and *I*4_1_/*acd* symmetry, marked by red arrows. (**b**) HRTEM: HR imaging of a garnet from Cazadero (corresponding [111] SAED pattern in the inset). The planar discontinuity is consistent with the occurrence of a twinning plane. Dark contrast around the twinning plane is due to local crystal structural strain.

The single-crystal XRD results are supported by electron diffraction tomography (EDT) experiments. Reconstructed diffraction volumes from seven areas from a garnet from the Cazadero blueschist show a pseudo-cubic cell with an identical orientation. However, in all cases one of the main cell vectors is systematically longer than the average value by an amount comparable to the EDT uncertainties (~2%) on cell parameters. This difference persists after the sample was rotated by 90°, excluding the possibility for experimental errors associated with the mechanical and optical alignment of the TEM. Thus, the EDT intensity distributions confirm a tetragonal symmetry. Moreover, violations on extinction conditions are observed in the reconstructed 3D diffraction volume, consistent with space group *I*4_1_/*a* (Fig. 6a; Supplementary Fig. S4). Ab-initio structure solution was achieved in space groups *Ia*-3*d*, *I*4_1_/*acd* and *I*4_1_/*a*. A tentative refinement of the distribution of Ca and Fe provided no evidence for short-range ordering.

Investigations using high-resolution transmitted electron microscopy (HRTEM) were performed mostly in the [111] orientation, but crystals were also tilted in order to highlight any TEM contrast and, thereby, assess any evidence for strain, twinning and structural defects. All garnets display a homogeneous, ordered and undeformed crystal structure, with very limited strain or defects. Selected area electron diffraction (SAED) patterns show intense and sharp reflections, with no evidence for spot splitting or streaking. Rarely, the ordered crystal structure displays isolated planar features (Fig. 6b) that may be consistent with the occurrence of twinning.

**Figure 7:**
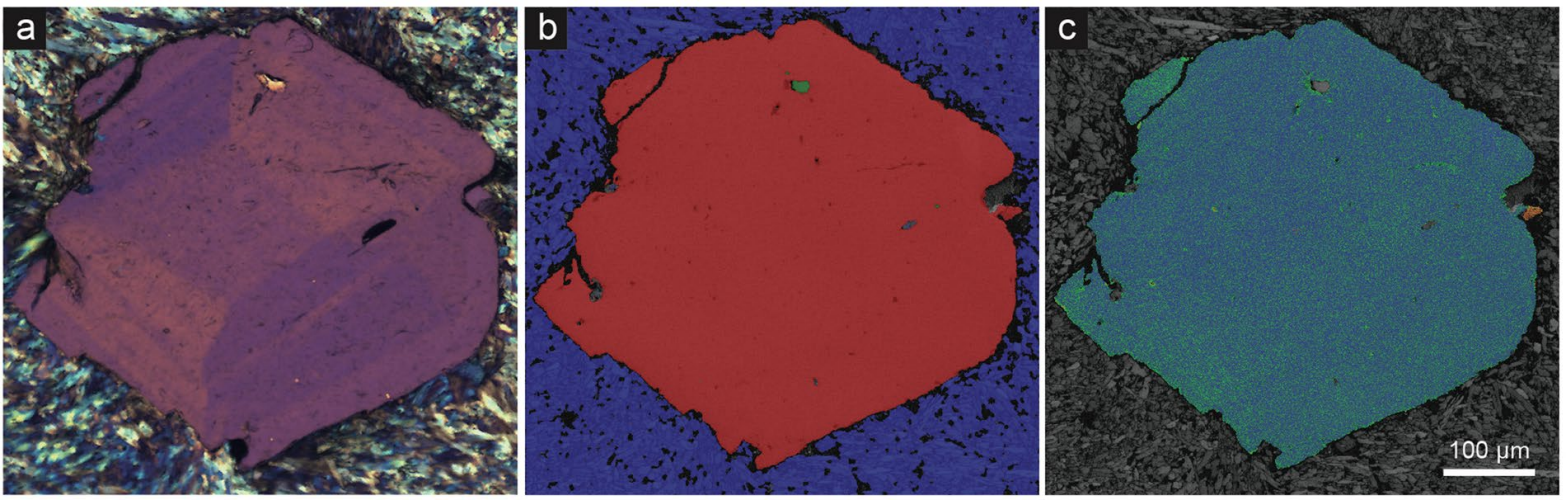
Petrographic and electron microscopic images of an anisotropic garnet from Cazadero. (**a**) Crossed-polarized optical photomicrograph of garnet showing well-developed sector zoning. (**b**) Phase recognition map from same field of view as (**a**) indexed with electron backscatter diffraction. Red = garnet; blue = glaucophane; green = quartz. (**c**) Texture component map (0–2.5°) with a rainbow color scheme reveals misorientation from the mean crystallographic orientation of the grain. Blue-green domains are close to the average orientation, while warm colors represent higher degrees of misorientation from the grain average. Apart from misorientation related to a fracture on top left, apparent misorientation from the grain average is an artefact due to changes in the unit cell parameters related to intracrystalline chemical variation.

Electron Backscatter diffraction (EBSD) mapping of garnet from the Cazadero blueschist does not reveal any significant microstructures that correlate with the observed optical sector zoning. This suggests that the diffraction patterns are equivalent across the sector boundaries, and that boundaries are merohedral twin planes^34^. At the micrometre scale, the grains are nominally undeformed with intragranular misorientation <2.5° across grains that are >600 µm in diameter. While minor misorientation is revealed in both the texture component and grain rotation hypermaps (Fig. 7; Supplementary Fig. S5), the close correlation between the misorientations with chemical variations resulting from the energy dispersive X-ray maps suggests that this is an artefact caused by chemically-controlled shifts in the *d* spacing rather than by true structural defects.

## Discussion

Our results clearly demonstrate the tetragonal nature of anhydrous Ca- and Fe^2+^-rich common garnet in the blueschists and phyllites investigated as part of this study. It may be argued whether these are exceptional features occurring in local and/or unusual environments or, rather, whether they are representative of the general behaviour of low-temperature metamorphic settings. We favour the latter interpretation for the following reasons: i) we have analysed samples from six widely-distributed geographical localities and which occur in well-known and diverse geological settings (the Franciscan complex, Alpine Corsica, the Southalpine and Austroalpine domains of the Alps); ii) we randomly chose our samples from these low-grade metamorphic complexes, and; iii) all other blueschists from Farinole contain optically sector-zoned garnets, regardless of their precise bulk composition. In this respect, it is striking that birefringent, twinned garnet was observed in lithologically-diverse samples at Cazadero, including metachert, meta-ironstone and metacarbonate^30^. Birefringent garnet is also present in the schists of the Sambagawa metamorphic belt, Japan (O. Weller, personal communication), and in the lawsonite–epidote blueschists of the Sivrihisar Massif, Turkey (D. Whitney, personal communication). In both occurrences^35,36^ the garnet has the grossular-rich (~25 mol%), pyrope-poor (<7 mol%) composition that typifies the samples documented here.

Reduction of symmetry in hydrated and anhydrous natural garnets has been observed by others, who similarly proposed tetragonal space groups *I*4_1_/*acd* or *I*4_1_/*a*. Two studies^21,37^ observed cell parameters with *c* > *a*, consistent with our results, whereas two others^38,39^ observed the opposite (*c* < *a*). The difference is probably connected with the Jahn–Teller distortion reported by the latter authors (refs^38^,^39^) for the octahedral Y sites. Such distortion, likely associated with the presence of Mn, induces an elongation along *a*. By contrast, Jahn–Teller distortion is not present in the structures reported by us or in other studies^21,37^, in which the octahedral sites are occupied by Al. In addition, our samples differ from those where *c* < *a* has been observed^38,39^ in their markedly different state of hydration and in the different Fe:Ca ratio, which results in a different size for the X sites. Therefore, we infer that in common natural garnets (i.e., anhydrous and dominated by Fe, Mg and Mn in the X site) that exhibit birefringence, the tetragonal condition is *c* > *a*, as observed in all samples investigated here.

In summary, a conclusion of the general applicability of the tetragonal nature of anhydrous common garnet in low-temperature metamorphic settings appears justified, and represents a hypothesis that is readily testable. Notably, there are no other studies demonstrating that common garnet from low-temperature metamorphic rocks and with a composition similar to those reported here are optically isotropic and have a cubic structure. We maintain that the non-cubic nature of these garnets has been overlooked because of the extremely low birefringence, whose detection is precluded in most cases due to examination of standard thin sections (30 μm) using a standard petrographic microscope^20^. We suggest that the use of thicker sections will reveal many more examples of birefringent, non-cubic garnets in low-grade metamorphic rocks worldwide.

We also suggest that garnet initially grows as a tetragonal mineral in most low-temperature metamorphic rocks. Instead of envisaging a symmetry decrease by phase transition upon slow cooling (i.e. a retrograde feature)^21^, we propose that the tetragonal structure occurs during growth under low-temperature metamorphic conditions (i.e. a prograde feature), as also observed in grossular-rich garnets from contact metasomatic and hydrothermal ore deposits^40^. Microstructural and petrological indications supporting a direct tetragonal growth, rather than an inversion from a cubic precursor, are the presence of sector twinning and the general lack of evidence for now tetragonal garnets having formed at higher *P–T* metamorphic conditions, from which they would have cooled/decompressed.

The Cazadero sample is particularly useful in constraining this low-*T* monometamorphic process, as the garnet preserves concentric and oscillatory growth zoning, and contains abundant inclusions of very low-*T* minerals, such as stilpnomelane and deerite (Figs 2a and 5), which are absent from the rock matrix. In addition, the sectored distribution of inclusions in garnet from Pfitscher Joch, which coincides with the optical sectors in the grain itself (Fig. 2c; Supplementary Fig. S1; Supplementary Videos S3 and S4), provides robust evidence that sector twinning is a growth feature^41^. The possibility that the tetragonal structure formed after any hypothetical lower symmetry precursor is not supported by our data. Instead, the occurrence of orthorhombic, monoclinic and even triclinic symmetries has so far been only been reported only for ‘uncommon’ garnet compositions, such as hydrogarnet and ugrandite^14,15^.

We argue that preservation of the tetragonal structure is favoured in rocks that were not metamorphosed to higher temperature, where a transition to the cubic form (and with a different chemical composition) would occur. The data from the Pfitscher Joch schist, which records the highest metamorphic temperatures of the sample studied (500–550 °C) and shows the closest approach to a cubic symmetry (Supplementary Table S2), supports this inference.

Concerning the possible *P–T* conditions at which the tetragonal–cubic inversion might take place, as observed experimentally in leucite^42^ and grossular^37^, we have performed annealing experiments on garnet crystals from Cazadero, both in an ambient-pressure furnace at 950 °C for 72 hours, and in a piston-cylinder at 1 GPa, 1000 °C for 232 hours. In both cases the recovered garnets remained anisotropic. These results suggest that the peculiar composition of the garnets documented in this study is resistant to inversion to a cubic structure, and that significant changes in garnet composition (increase of pyrope and decrease of grossular components) are required for such an inversion.

The lowering of symmetry observed here would normally be interpreted as the result of the partitioning of the larger Ca and smaller Fe^2+^, Mg and Mn cations within non-equivalent *X* sites in the structure, as proposed (although not proven) in earlier studies^21^. Thermodynamic investigation of the mixing properties along the grossular–almandine binary has suggested the occurrence of an ordered compound with composition Fe_2_CaAl_2_(SiO_4_)_3_, with a Ca–Fe ratio similar to that measured in the tetragonal garnets studied here^43^. However, the results of our XRD and EDT analyses do not provide evidence for ordering, either short- or long-range, of divalent cations in the garnet structure. Therefore, the cause of the tetragonal symmetry remains an open question that requires further investigation, possibly with the aid of spectroscopic techniques^14^.

Investigating the possible causes and processes arising from cation partitioning is “challenging but vital”^14^ for the major implications of non-ideality on the thermodynamic properties of garnet solid solution^44^, on the stability and composition of this mineral during metamorphism, on inter- and intracrystalline diffusional processes and their applications^45^, and on the behaviour of synthetic non-silicate garnet used in technological applications^14^. It follows that the tetragonal nature of crystals grown under low-grade metamorphic conditions requires a reconsideration of the crystal-chemical and thermodynamic properties of garnet. Due to the wide pressure–temperature–composition stability field of garnet, the latter are key for constraining the pressure–temperature–time history of rocks from the shallow crust to deep lithosphere, from which large-scale tectonic processes may be inferred. Using incorrect thermodynamic properties and mixing models for a relevant metamorphic mineral such as garnet affects our ability to model the geodynamic processes attending metamorphism, although the magnitude of such effects awaits further investigation.

**Figure 8:**
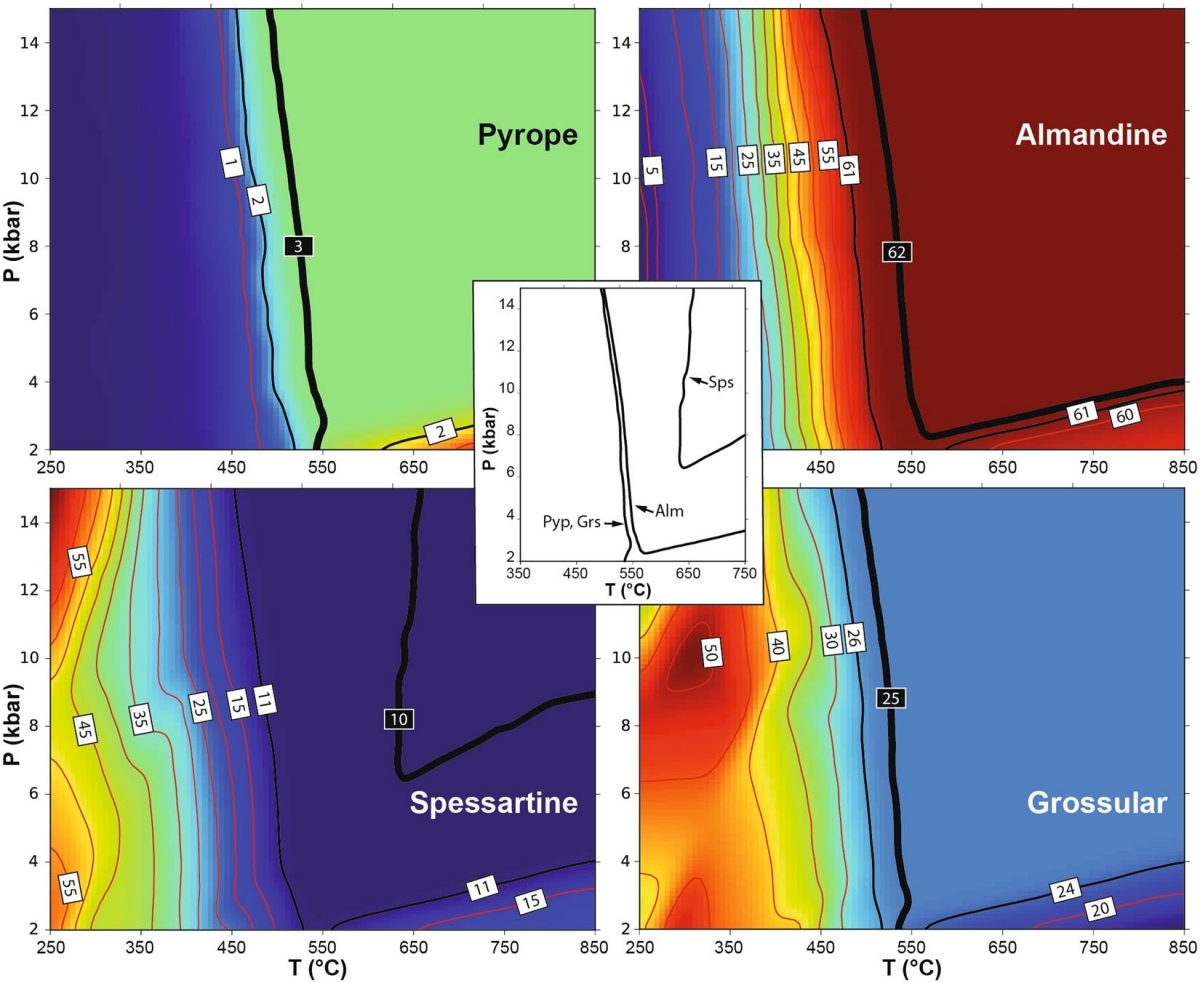
Thermodynamic calculation of the stability and composition of garnet. The model used coincides with an Alm_62_Grs_25_Sps_10_Pyp_3_ garnet composition in the SiO_2_–Al_2_O_3_–FeO–MnO–MgO–CaO–H_2_O system and assumes the presence of a saturating pure H_2_O volatile phase. Background color ranges from dark blue (low concentrations) to red (high concentrations). Thin red lines are isopleths, labelled in mol%, of the garnet components considered in each panel (from top left counterclockwise pyrope, almandine, grossular and spessartine). Thick black lines are isopleths of the target (input) composition, reported within black labels. Thin black lines are isopleths of composition corresponding to ±1 mol% of the input composition. Central inset reports the P-T location of the target isopleths.

The possible limitations of thermodynamic parameters pertaining to the onset of garnet growth at low temperature have already been highlighted^46^, and could explain why current mixing models in some cases fail to predict the presence of garnet in low-*T* metamorphic rocks^3,47^. As the lack of stability of one phase could also be due to the use of an inappropriate effective bulk composition, we performed a simple test by analysing the stability and composition of garnet in a model SiO_2_–Al_2_O_3_–FeO–MnO–MgO–CaO–H_2_O system, coinciding with a typical tetragonal garnet composition measured in this work at Cazadero (Alm_62_Grs_25_Sps_10_Pyp_3_, see Supplementary Table S1), assuming the presence of a pure H_2_O volatile phase that permits the stabilization of hydrous phases. The results of phase equilibria modelling in the range 250–850 °C and 0.2–1.5 GPa, performed with the Perple_X software^48^ and using the most recent thermodynamic dataset and solution models^49^, are reported in Fig. 8. The calculations show that the target (input) garnet composition is stable within an uncertainty of ±1 mol% at *T* > 500 °C, and that temperature has to be almost 650 °C to match exactly the input composition. In this respect, the predicted concentration of the spessartine component shows the poorest match for that in the natural garnet composition. The results of the modelling (not shown) do not differ significantly when a mixed H_2_O–CO_2_ fluid is present to account for low *a*_H2O_ conditions.

The temperature conditions of garnet stability (>500 °C) predicted by the modelling contrast with the much lower temperatures (<350 °C) proposed in the literature^23^, which are supported by an inclusion assemblage including deerite, chlorite and stilpnomelane. This test reinforces the perspective that existing thermodynamic properties of garnet that grows in the initial stages of subduction and in the lowest-grade part of orogenic belts may need to be re-assessed. The characterization of natural samples with a tetragonal structure, such as in this study, may be used to refine the calorimetric properties and mixing parameters of garnet at low-*T*.

## Methods

### Materials

Polished regular (30 μm thick) thin sections and doubly polished 100 to 200 μm thick sections were obtained from the six studied samples of blueschists, phyllite and schist.

In addition, single crystals of garnet were handpicked after selective High Voltage Pulse Fragmentation using Selfrag AG Lab system. 200 μm thick doubly-polished equatorial wafers of single crystals were then prepared for optical observation.

### Electron Microprobe Analysis (EMPA)

The major element composition of garnet and the X-ray elemental maps were obtained with a Jeol JXA 8200 Superprobe at the Dipartimento di Scienze della Terra, Università di Milano, Italy. Analytical parameters were 15 kV accelerating voltage, 5 nA current, counting time of 30 s on peak and 10 s on background.

### Fourier-Transform Infrared Spectroscopy (FTIR)

Single crystals from Cazadero blueschist and from Pfitscher Joch and eastern Alps phyllites were manually separated from the rocks and doubly polished such as to obtain an equatorial slice through the garnet, with thickness ranging between 325 and 315 μm. FTIR raster maps^50^ were acquired with aperture 40 × 40 μm^2^ using a Bruker Hyperion 3000 microscope equipped with a KBr broadband beam splitter, a 15X objective and a liquid nitrogen-cooled MCT detector at Istituto Nazionale di Fisica Nucleare (LNF- INFN), Frascati, Italy. A conventional (Globar) source was used for the IR beam; the nominal resolution was set at 4 cm^−1^ and 128 scans were averaged for both spectrum and background. High-resolution FTIR images were collected with a 64 × 64 pixel focal-plane array (FPA) of liquid nitrogen-cooled MCT detectors, coupled to a 15X Schwarzschild objective. The nominal resolution was set at 8 cm^−1^ and 64 scans were averaged for each spectrum and background; in these conditions each image covers an area of 170 × 170 μm, with a spatial resolution of ~5 μm^51^. The final images were obtained as a mosaic of several single images, displacing the sample along a grid at 170 μm step, such as to cover the desired area.

### Electron Backscattered Diffraction (EBSD)

Thin sections of samples from Cazadero and Farinole were prepared with a mechanical polish using 1 µm diamond paste. After garnet grains were identified and imaged optically the section was given a final chemical-mechanical polish using 50 nm colloidal silica dispersion in NaOH. After polishing, garnets were imaged using backscattered electron (BSE) atomic contrast imaging with a Tescan Mira3 field emission gun scanning electron microscope (FEG-SEM). Two garnet grains were then mapped by electron backscatter diffraction (EBSD). Electron backscatter patterns (EBSPs) were collected from the garnets and the surrounding matrix in orthogonal grids using a Nordsly Nano high resolution detector and Oxford Instruments Aztec 2.4 acquisition software package on the Mira3 FEG-SEM. EBSD analyses were collected with a 20 kV accelerating voltage, 70° sample tilt, ~20 mm working distance, and 18 nA beam current. EBSPs were collected with the following parameters; an acquisition speed of ~40 Hz, 64 frames were collected for a background noise subtraction, 4 × 4 binning, high gain, a Hough resolution of 60, and band detection min/max of 6/8. Maps were collected in orthogonal grids with a step size between 2.0 µm and 2.5 µm. Mean angular deviation values of the electron backscatter patterns for the maps ranged between 0.36 and 0.35. Individual garnet grains were indexed using the Grossular match unit based on the unit cell parameters of 1 with a = b = c = 11.8451 Å. Glaucophane of the surrounding matrix was indexed using the match unit Glaucophane after the unit cell parameters of 2. Additional matrix and inclusion minerals are apatite and quartz. Apatite was indexed using the oxyapatite match unit from the cell parameters of 3, and quartz was indexed using the Quartz new match unit based on the unit cell parameters of 4.

Post-processing the EBSD data was undertaken with Oxford Instruments Channel 5.11 software suite. All EBSD data was given a wild-spike noise reduction and a seven nearest neighbour zero-solution correction, no other corrections were applied. Using Tango suite of Channel 5 the following EBSD maps were produced:

1. Colored phase maps, garnet is blue, glaucophane is green, quartz is red, and apatite is yellow;
2. All Euler crystallographic orientation map;
3. Texture component maps (0–2.5°), colored with a rainbow scheme revel misorientation from the mean orientation of the grain as determined using the grain detect function of Tango. Blue domains are close to the average orientation, while warm colors represent higher degrees of misorientation from the grain average. Apparent misorientation from the grain average is due to changes in the unit cell parameters related to intragranular chemical variation.
4. Grain misorientation map, using the grain rotation orientation direction (GROD)-hyper function of Channel5, which helps visualize the substructure of the grains by plotting the deviation angle of each pixel and the disorientation axis from the mean grain orientation, grain boundaries are defined as >10°.

Simultaneous with EBSD mapping, energy dispersive X-ray spectra were collected from each pixel. The X-ray spectra for the maps were collected using an Oxford instruments X-Max detector and were processed using the Aztec 2.4 software package.

### High-Resolution Transmission Electron Microscopy (HRTEM)

TEM investigations were performed by a JEOL 2010 microscope, working at 200 kV, with ultra-high resolution (UHR) pole piece and point-to-point resolution of 0.19 nm. The microscope is equipped with semi-STEM control and ultra-thin window energy dispersive spectrometer (EDS ISIS Oxford). Data were recorded by an Olympus Tengra CCD camera (2k × 2k × 14 bit). Sample preparation has been done by Ar+ ion milling (Dual Ion Mill Gatan and PIPS Gatan 691). TEM grids were extracted from polished petrographic sections, selecting at least two birefringent garnets for each sample (AUS, FRAN and SPIA). Ion milling had two main consequences: on one side, the obtainment of ultrathin samples (less than 100 nm) significantly reduced the garnet birefringence colour in the petrographic microscope, providing almost extinct crystals; on the other side, many crystallographic features, observed in the 30 μm thick samples, were no longer visible, thus complicating the identification of features such as sector boundaries, possible twinning planes or mottled pattern.

### Electron diffraction tomography (EDT)

EDT data collections^52–54^ were performed by a Zeiss Libra TEM operating at 120 kV and equipped with a LaB_6_ source and a Bruker EDS detector XFlash6T-60. EDT acquisitions were done in STEM mode after defocusing the beam. A beam size of about 150 nm in diameter was obtained by inserting a 5 μm C2 condenser aperture. EDT data were recorded by a background-free single-electron ASI Timepix detector^55^, which allows an extremely mild illumination thus preventing any alteration or amorphization of the sample.

EDT data sets were acquired with and without precessing the beam. Precession was obtained by a Nanomegas Digistar P1000 device, with a semi-angle of about 1°. EDT data used for dynamical structure refinement were acquired with precessing beam for better reflection intensity integration, in tilt steps of 1° for a total tilt range of −60°/+60°. A camera length of 180 mm was used, equivalent to a resolution of about 0.75 Å. Data analysis, including cell parameter determination, reflection intensity integration, ab-initio structure determination and dynamical refinement^56^, was performed by the PETS-JANA package.

EDT intensity distribution suggest again a tetragonal symmetry. Moreover, clear violations on extinction conditions are observed in the reconstructed 3D diffraction volume, consistent with space group *I*4_1_/*a* (Fig. 6a, Supplementary Fig. S4). Ab-initio structure solution was achieved in space groups *Ia*-3*d*, *I*4_1_/*acd* and *I*4_1_/*a*. Residuals obtained by dynamical refinement in these space groups are comparable, with differences of only 1% in R1. The two larger crystallographic sites occupied by Fe and Ca appear quite similar, both in terms of Fe/Ca-O interatomic distances and in terms of geometry. A tentative refinement of the distribution of Ca and Fe resulted in a comparable partial occupancy for the two sites.

### Single-Crystal X-Ray Diffraction (XRD)

A birefringent garnet from Jenner was investigated by single-crystal X-ray diffraction using two different X-ray diffractomers. Using a point detector diffractometer controlled by the SINGLE software^57^, which is designed to apply the 8-centering positions, allowed to measure the unit-cell parameters with very high accuracy and precision. We collected 45 different reflections (each measured in 8 different positions) and the results (Supplementary Table S2) clearly indicate a tetragonal symmetry with a = b = 11.6064(4) Å, c = 11.6146(8) Å, volume = 1564.59(13) Å^3^. The difference between *a, b* and *c* axes is well above 10 times the experimental uncertainty and thus the tetragonal cell is definitively reliable. The c/a ratio = 0.0007 is in agreement with the value of 0.0009 recently determined for a hydrous natural tetragonal garnet.

The same crystal was then studied using a second single-crystal diffractometer to collect complete intensity data and obtain structural data. We have measured a spherical shaped crystal with an average radius of 0.08 mm up to 2θ = 82° and collected the full Ewald sphere summing a total of 47660 reflections of which 1279 unique (Rint = 0.045). WINGX package and SHELX software^37,58,59^ were used for structure refinement. The systematic absences and the statistical analysis of the intensities are consistent with *I*4_1_/*acd* space group and the structure (see Supplementary Table S2) was refined starting from recently published structural models^17,37,38^. Our model does not report the F11 and O22 sites (see^17^) because the investigated garnet is anhydrous and does not contain any F. However, our structure refinement shows an excellent agreement factor with R1 = 0.021 to indicate that the space group here adopted is absolutely reliable. This agreement factor is better, or at least comparable, with those obtained for previous refinements on natural hydrous tetragonal garnets which show the same *I*4_1_/*acd* space group as in our work. In terms of site occupancy, Fe, Ca, Mn and Mg occupy the X1 and X2 crystallographic sites and we do not observe any site preferences among such elements within these sites. This is definitively confirmed by the X1-O an X2-O average bond distances, which are practically identical within one uncertainty (e.g. Ca, for example, has a much longer cation radius with respect to the other ions and this would be strongly evident on the bond distances in case of Ca preference for one of the two sites). All crystallographic information are deposited with the CIF file (Supplementary Data S1).

## Supplementary information

Supplementary Figures and Tables

Video S1

Video S2

Video S3

Video S4

Supplementary Data S1

## Data Availability

The datasets generated during and/or analysed during the current study, whether not included in this published article and its Supplementary Information files, are available from the corresponding author on reasonable request.
