# Supplementary material for: Garnet, the archetypal cubic mineral, grows tetragonal
Source: Sci Rep. 2019 Oct 11;9:14672. doi: 10.1038/s41598-019-51214-9 (PMC6789019; doi:10.1038/s41598-019-51214-9)
Supplement: Supplementary file 1 — Supplementary Figures and Tables [file 41598_2019_51214_MOESM1_ESM.pdf]

Garnet, the archetypal cubic mineral, grows tetragonal

(Supplementary Material Online)

Cesare B.[1*], Nestola F.[1], Johnson T.[2], Mugnaioli E.[3], Della Ventura G.[4,5], Peruzzo L.[6], Bartoli O.[1], Viti C.[7], Erickson T.[8]

1) Dipartimento di Geoscienze, Università degli Studi di Padova, via Gradenigo 6, 35131 Padova, Italy
2) School of Earth and Planetary Sciences, Curtin University, Bentley 6102, Perth, Australia
3) Center for Nanotechnology Innovation@NEST, Istituto Italiano di Tecnologia, Piazza San Silvestro 12, 56127 Pisa, Italy
4) Dipartimento di Scienze, Università di Roma Tre, Largo San Leonardo Murialdo 1, 00146 Rome, Italy
5) Istituto Nazionale di Fisica Nucleare, Via Enrico Fermi 40, 00044 Frascati, Italy
6) Istituto di Geoscienze e Georisorse, CNR, via Gradenigo 6, 35131, Padova, Italy
7) Dipartimento di Scienze Fisiche, della Terra e dell'Ambiente, Università di Siena
8) Jacobs – JETS, NASA Johnson Space Center, Astromaterials Research and Exploration Science Division, Mailcode XI3, 2101 NASA Parkway, Houston, TX, 77058 USA

*) corresponding author: bernardo.cesare@unipd.it

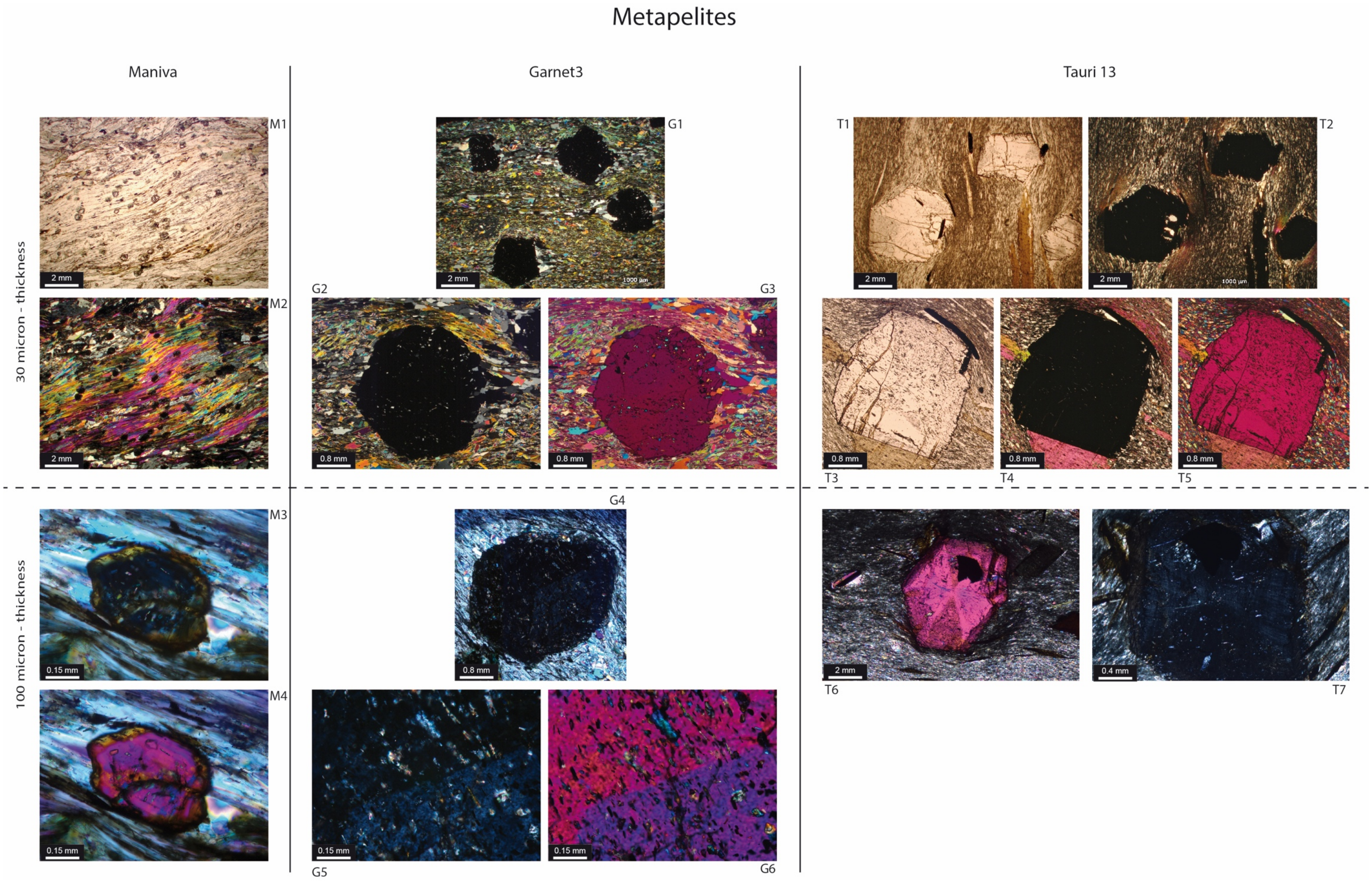

Fig. S1- Petrographic features of the studied metapelitic rocks, with emphasis on optical anisotropy of garnets as evidenced comparing regular 30-µm and thicker 100-µm sections.

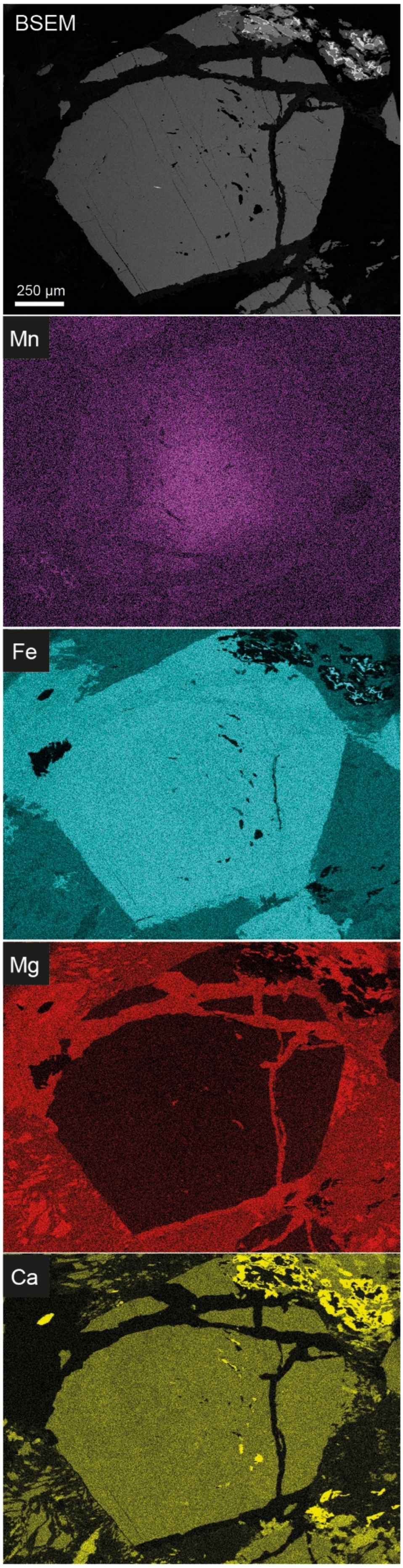

Fig. S2 - Backscattered electron SEM (BSEM) image and X-ray map of the distribution of Ca, Fe, Mn and Mg in a garnet from Farinole, showing a very weak bell-shaped zoning of Mn in an otherwise flat compositional profile.

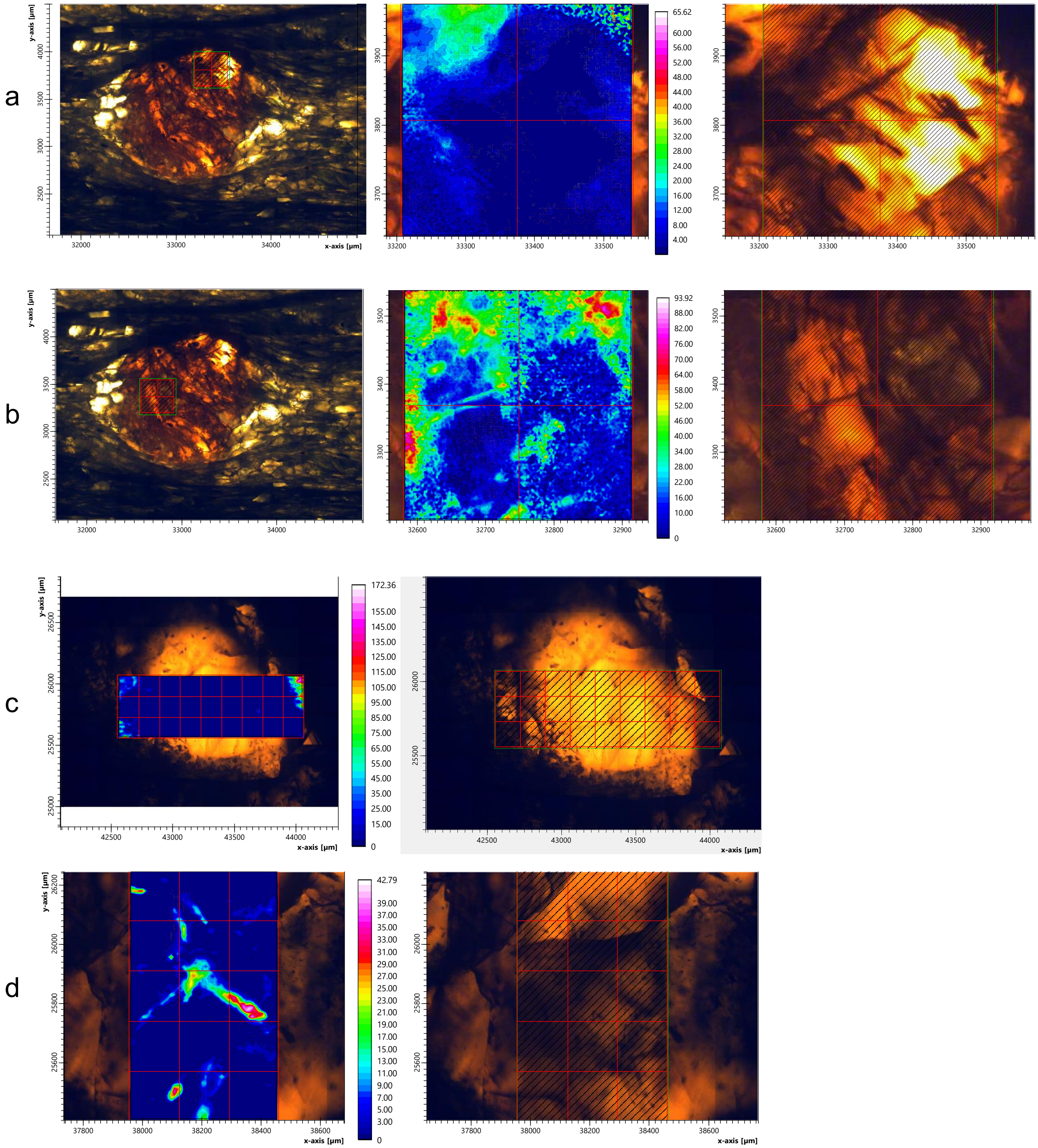

Fig. S3 - FTIR images of some selected garnets from metapelitic rocks. (a) and (b): Pfitscher Joch. From left to right are the locations of targets in the garnet, the mosaic of four FPA frames each, and the detailed microscopic view of the target in transmitted light. (c) and (d): Unknown locality in the eastern Alps. From left to right are two targets consisting of a mosaic of 27 and 15 FPA frames, respectively, and their detailed microscopic view in transmitted light microscopy. All examples, in particular (c) and (d), clearly show that the garnets are systematically H_2O free, while a very low amount of H_2O is exclusively associated with the mineral inclusions within the garnet host.

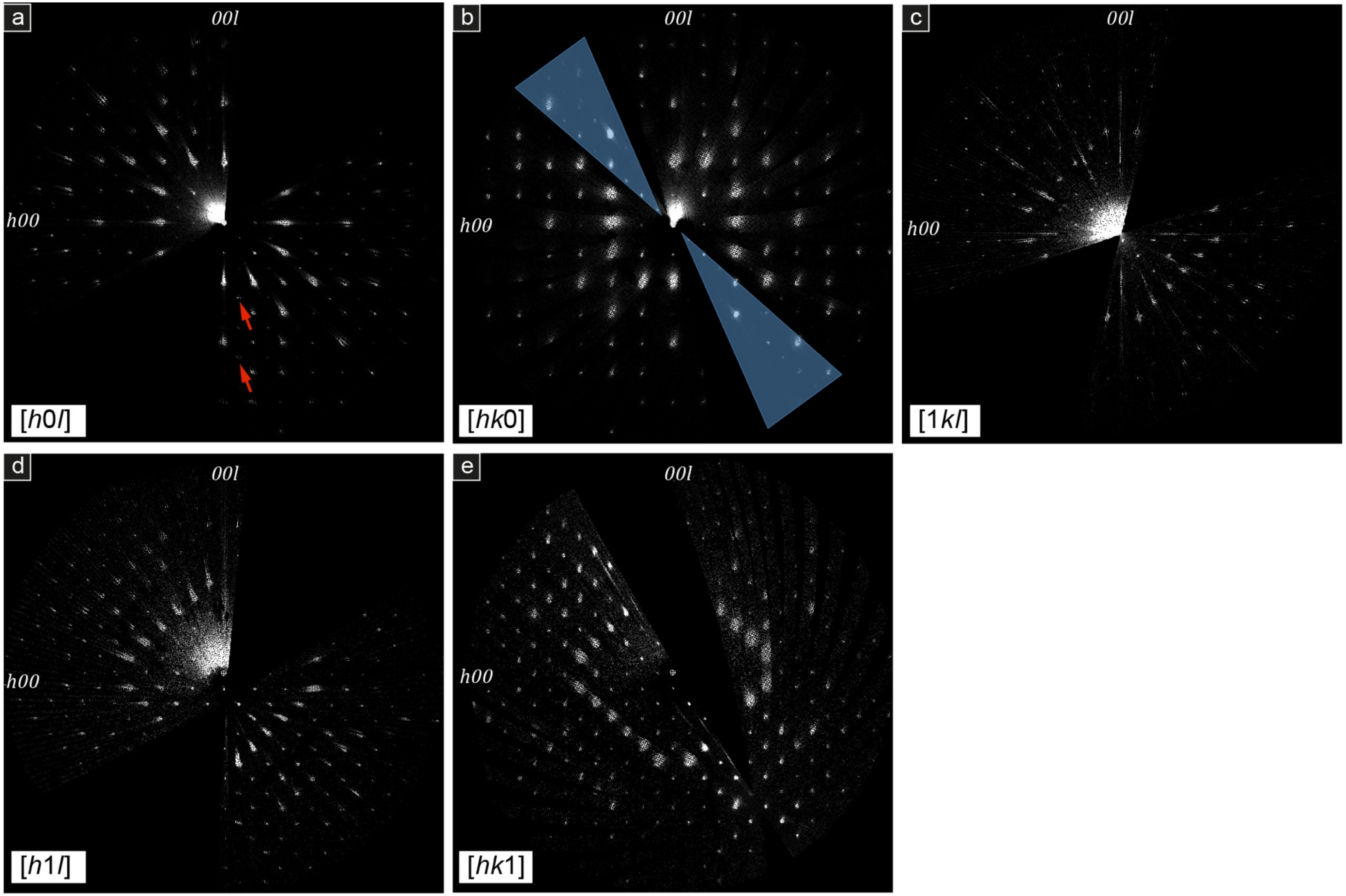

Fig. S4 - Sections of the 3D diffraction volume obtained by EDT data. **a.** *h*0*l* plane, showing reflections *h*0*l* : *h*,*l* ≠ 2*n* marked by red arrows. **b.** *hk*0 plane, showing only reflections 0*kl* : *k*,*l* = 2*n*; some violated reflections appearing only close to the tilt axis track (in the blue triangular fields) are to be considered artefacts of the reconstruction. **c.** 1*kl* plane, with the whole 1*k*0 reflection row extinct. **d.** *h*1*l* plane, with the whole *h*10 reflection row extinct. **e.** *hk*1 plane, with no extra extinction beside the one derived by the body centered lattice. Reconstructions are made by PETS software.

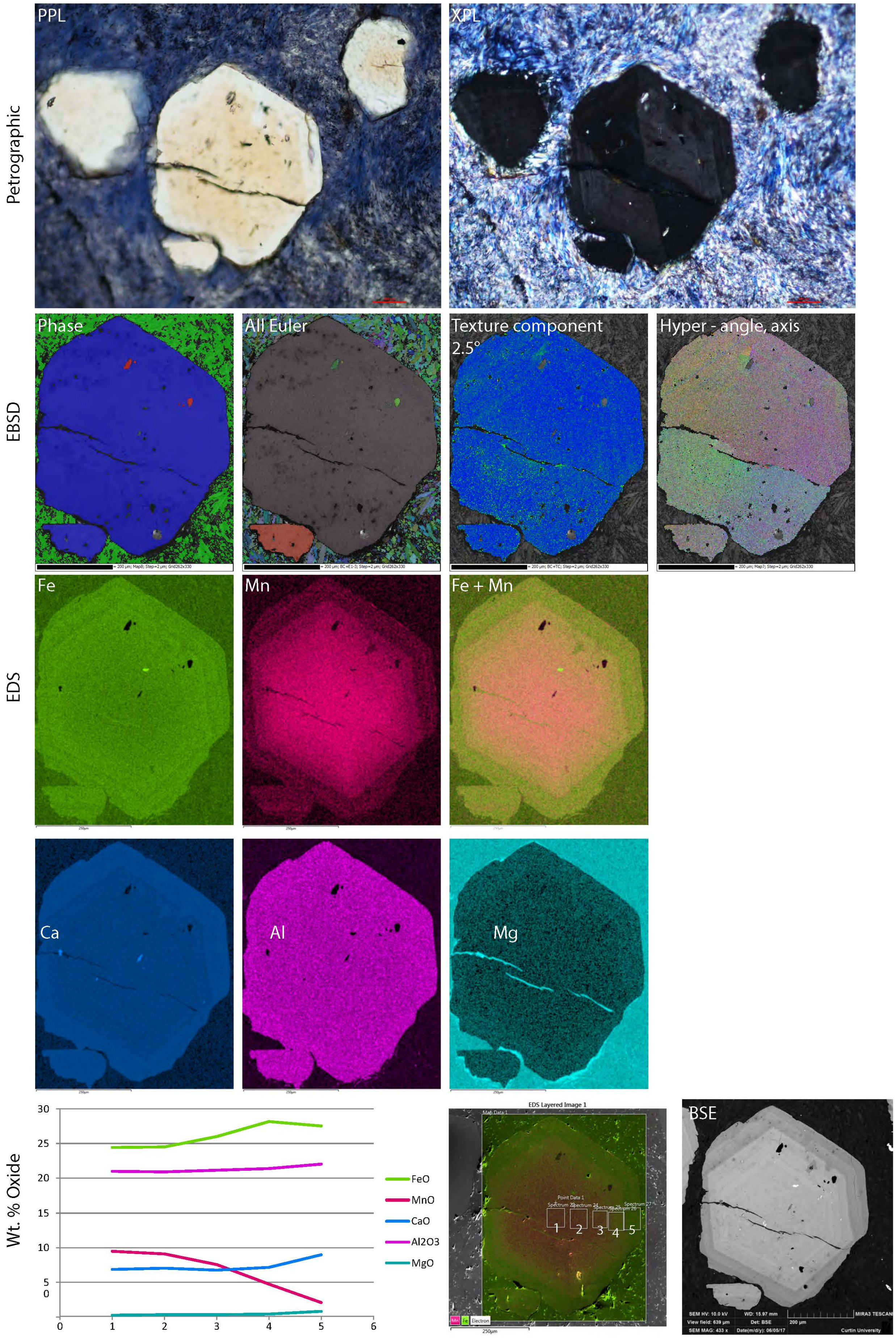

Fig. S5 - Optical micrographs, electron backscatter diffraction micrographs (EBSD), X-ray maps of the distribution of major elements, compositional core (left) - rim (right) profile, and backscattered electron SEM (BSE) image of an optically birefringent, sector-zoned garnet from Cazadero

Sample	Jenner				Cazadero				Farinole				East. Alps		Maniva		Pfitscher J.	
position(1)	c	r	c	r	c	r	c	r	r	c	r	c	r	c	c	r	c	r(2)
MgO	1,19	1,39	1,08	1,29	0,28	0,97	0,30	0,71	2,00	0,73	1,72	1,03	1,78	0,90	1,32	2,16	1,40	2,94
Al_2O_3	20,61	20,79	20,85	21,22	19,75	21,03	18,98	20,65	21,36	20,73	20,95	20,73	21,01	20,53	21,39	21,44	21,34	21,21
SiO_2	36,75	37,42	37,59	36,99	36,52	36,62	38,68	36,62	36,98	37,10	37,22	36,80	36,89	36,95	37,05	36,94	37,38	37,36
K_2O	0,20	0,13	0,14	0,14	0,00	0,20	0,01	0,02	0,00	0,00	0,00	0,00	0,00	0,00	0,01	0,00		
CaO	9,97	11,25	11,57	10,23	9,27	10,13	6,75	9,84	8,51	8,97	7,99	9,05	5,90	6,82	6,75	4,05	8,12	4,60
TiO_2	0,21	0,15	0,20	0,10	0,03	0,14	0,01	0,14	0,10	0,18	0,07	0,17	0,06	0,12	0,05	0,06	0,07	0,02
MnO	3,81	0,43	0,76	0,55	8,43	3,27	9,22	1,76	0,13	4,60	0,33	2,98	0,97	5,96	2,08	0,27	4,25	0,72
FeO	27,60	29,30	29,27	30,58	26,35	27,85	26,16	30,37	31,93	28,95	32,94	30,81	34,01	29,06	32,25	36,04	28,54	34,23
Total	100,34	100,86	101,46	101,10	100,62	100,21	100,10	100,13	101,02	101,26	101,23	101,57	100,61	100,34	101,16	100,99	101,10	101,07
Si	2,957	2,976	2,976	2,946	2,971	2,947	3,130	2,960	2,943	2,969	2,967	2,944	2,966	2,988	2,963	2,958	2,974	2,973
Ti	0,012	0,009	0,012	0,006	0,002	0,008	0,001	0,009	0,006	0,011	0,004	0,010	0,003	0,007	0,003	0,003	0,004	0,001
Al	1,955	1,949	1,946	1,992	1,893	1,994	1,810	1,967	2,003	1,955	1,969	1,954	1,991	1,957	2,016	2,024	2,001	1,990
Fe2+	1,857	1,949	1,938	2,037	1,793	1,874	1,770	2,053	2,125	1,938	2,196	2,061	2,287	1,966	2,157	2,414	1,899	2,278
Mn	0,260	0,029	0,051	0,037	0,581	0,223	0,632	0,121	0,009	0,312	0,022	0,202	0,066	0,408	0,141	0,018	0,286	0,048
Mg	0,143	0,165	0,128	0,153	0,034	0,117	0,036	0,086	0,237	0,087	0,204	0,123	0,213	0,109	0,158	0,258	0,166	0,349
Ca	0,859	0,958	0,981	0,873	0,808	0,873	0,585	0,852	0,726	0,770	0,682	0,775	0,508	0,591	0,579	0,348	0,692	0,392
Total	8,043	8,034	8,032	8,044	8,081	8,037	7,964	8,047	8,049	8,042	8,044	8,069	8,035	8,026	8,052	8,027	8,021	8,031
Alm%	59,5	62,8	62,6	65,7	55,8	60,7	58,5	66,0	68,6	62,4	70,7	65,2	74,4	64,0	71,1	79,5	62,4	74,3
Pyp%	4,6	5,3	4,1	4,9	1,1	3,8	1,2	2,7	7,7	2,8	6,6	3,9	6,9	3,5	5,2	8,5	5,4	11,4
Sps%	8,3	0,9	1,6	1,2	18,1	7,2	20,9	3,9	0,3	10,0	0,7	6,4	2,1	13,3	4,7	0,6	9,4	1,6
Grs%	27,5	30,9	31,7	28,2	25,1	28,3	19,4	27,4	23,4	24,8	22,0	24,5	16,5	19,2	19,1	11,5	22,7	12,8

	Range (3)	Range (core-rim)	Range (core-rim)	Range (core-rim)	Range (core-rim)	Range (core-rim)
Alm%	*65*	*58 65*	*70 75*	*65 74*	*71 79*	*62 75*
Grs%	*30*	*20 27*	*20 20*	*19 17*	*19 11*	*23 12*
Pyp%	*5*	*1 4*	*1 5*	*3 7*	*5 9*	*6 11*
Sps%	*1*	*21 4*	*9 0*	*13 2*	*6 1*	*10 2*

Table S1 - Representative EMP analyses of Garnet from all studied samples. Mineral formula recalculated based on 12 oxygens and all Fe as FeO. (1) location within crystals: c=core; r= rim; (2) rim of Pfitscher Joch garnet, showing the highest Mg and lowest Ca contents; (3) Garnet in blueschist from Jenner does not show significant core-rim zoning.

	**Sample**	***a* axis (Å)**	***c* axis (Å)**	**Δ (*c*-*a*)**	**Volume (Å^3)**	**Total reflections**	***c/a* ratio**	**Symmetry**
Bluesch.	Cazadero	11.6370(1)	11.6443(1)	0.0073(2)	1576.87(2)	14675	1.00063(1)	$I4_1/acd$
	Farinole	11.6064(4)	11.6146(4)	0.0082(8)	1564.59(14)	45*	1.00071(4)	$I4_1/acd$
Pelite	Pfitscher	11.6048(1)	11.6064(2)	0.0016(3)	1563.06(3)	24995	1.00014(2)	$I4_1/acd$
	Maniva	11.5606(1)	11.5721(2)	0.0115(3)	1546.58(3)	23589	1.00099(2)	$I4_1/acd$
	East. Alps	11.5935(1)	11.5987(2)	0.0052(3)	1558.97(3)	20828	1.00045(2)	$I4_1/acd$

*these unit-cell parameters were measured using a point detector and the software SINGLE (Angel and Finger 2011) and the 8-positions centering procedure (see text).

Table S2 - List of all samples investigated by single-crystal XRD. Columns report the *a* and *c* crystallographic axes, their absolute difference (in Angstrom), the unit cell volume, the number of total reflections measured, the cell parameter ratio and the space group symmetry. Parentheses report the values of one experimental uncertainty.

Data S1 - CIF file with full crystallographic information obtained from single-crystal XRD analysis of a birefringent garnet from Farinole blueschist.

Video S1 - Optical anisotropy in garnet from Cazadero. Crossed polarizers. Thickness of crystal approximately 150 µm. The video shows the exceptionally developed sector zoning, and the inclusion-rich garnet core. Width of view: 3.5 mm.

Video S2 - Optical anisotropy in garnets from Cazadero. Crossed polarizers and red tint plate to highlight birefringence. Thickness of crystals approximately 150 µm. The video shows the exceptionally developed sector zoning, and the inclusion-rich garnet cores. Width of view: 5.3 mm.

Video S3 – Optical anisotropy in garnet from a schist from Pfitscher Joch. Crossed polarizers, thickness of thin section approximately 100 µm. The video shows a well-preserved optical sector zoning despite the rock reached c. 550 °C. Width of view: 5.3 mm.

Video S4 –Close-up movie of the garnet from Video S3, showing both the optical sector zoning and the mottled pattern of birefringence within each sector. Width of view: 1.7 mm.
